# Zeolitic imidazolate frameworks (ZIF-8) as a carrier in a topical ocular delivery system for the treatment of ocular diseases

**DOI:** 10.1371/journal.pone.0346473

**Published:** 2026-04-21

**Authors:** Phillip A. Harding, James H. Westbay, Ethan Fletcher, Xin Fan, Morgan V. DiLeo

**Affiliations:** 1 Department of Bioengineering, Swanson School of Engineering, University of Pittsburgh, Pittsburgh, Pennsylvania, United States of America; 2 Department of Ophthalmology, School of Medicine, University of Pittsburgh, Pittsburgh, Pennsylvania, United States of America; 3 Department of Biomedical Engineering, School of Engineering, University of Mount Union, Alliance, Ohio, United States of America; 4 Clinical and Translational Science Institute, University of Pittsburgh, Pittsburgh, Pennsylvania, United States of America; 5 Department of Chemical Engineering, Swanson School of Engineering, University of Pittsburgh, Pittsburgh, Pennsylvania, United States of America; 6 McGowan Institute for Regenerative Medicine, Pittsburgh, Pennsylvania, United States of America; Beijing University of Chemical Technology, CHINA

## Abstract

Current systems for topical ocular drug delivery enable non-invasive administration of therapeutics to the eye but suffer from poor permeability and bioavailability, limiting therapeutic efficacy. Metal-organic frameworks are porous, crystalline materials that can serve as drug delivery systems and may improve these shortcomings. In this study, we explore the use of ZIF-8 as a representative metal-organic framework for topical ocular drug delivery. ZIF-8 particles were prepared via a simplified aqueous synthetic method and loaded with ovalbumin, a model molecule to simulate anti-angiogenic proteins that are used to treat retinal diseases. The physical properties of unloaded and loaded ZIF-8 were evaluated with a variety of spectroscopic, imaging, and desorption techniques that confirmed successful loading with ovalbumin (>46% loading capacity) without severe disruption to the structure of ZIF-8. In bulk solution, loaded ovalbumin is gradually released in a linear fashion for up to 60 days. Loaded ZIF-8 applied to chicken eggs or bovine eyes showed minimal to no levels of irritation, supporting our hypothesis that ZIF-8 can be safely applied to ocular tissue. When applied to ex vivo bovine eyes, loaded agents administered in ZIF-8 penetrate the sclera, suggesting ZIF-8 may enhance permeability to intraocular tissue. These results highlight the potential of ZIF-8 as a carrier in topical ocular delivery systems and provide a crucial foundation to evaluate their efficacy in topical ocular drug delivery using in vivo models.

## Introduction

There is a need to explore novel methods for ocular drug delivery that address the challenges presented by the ocular anatomy and physiological barriers, limiting drug bioavailability and therapeutic efficacy [1–4]. For posterior segment diseases, such as age-related macular degeneration (AMD) and diabetic retinopathy (DR), treatments involve invasive, surgeon-directed injections of anti-vascular endothelial growth factors (anti-VEGF) that have incidences of associated detrimental effects on retinal health and integrity such as ocular hypertension (occurs in all cases), ocular hemorrhaging (10% of patients), and retinal detachment (5–27% of patients) [5]. As a result, patient noncompliance rates can be seen up to 50% [6,7]. The use of nanoscale materials in either topical or injectable formulations of ocular therapeutics can potentially address the main issues of ophthalmic drug delivery: retention, permeability, and bioavailability [8,9].

Metal-organic frameworks (MOFs) are a repurposed class of crystalline nanoparticles with significant potential in ocular drug delivery. Previous drug delivery research has demonstrated that a wide range of MOFs can target and deliver therapeutic cargo [10–12], and have several advantages over polymeric nanoparticles, with surface areas up to ~7,000 m^2^/g, uniformly distributed pore volumes of ~4.40 cm^3^/g, structural and functional tunability by interchangeability of nodes and linkers, improved stability in solutions, and intrinsic theranostic capabilities [13–15]. This allows MOFs to encapsulate proteins at larger loading capacities (~40 wt % v 26 wt%) and demonstrate slower release of protein in different physiological environments [16]. Of particular interest for drug delivery are zeolitic imidazolate frameworks (ZIFs), specifically ZIF-8. ZIF-8 is a type of MOF synthesized from the coordination of Zn^2+^ metal nodes and 2-methylimidazole linkers, and they are widely considered to be a good drug delivery candidate due to their high loading capacity, biocompatibility, and stimuli-responsive release kinetics [17–19]. In addition, ZIF-8 can be synthesized via different methods including solvothermal, ultrasound-assisted, mechanochemical, and aqueous methods, with minimal differences between particle morphology purity, allowing for simple synthesis and increased biocompatibility [20,21].

In this study, we evaluated ZIF-8 as a potential delivery vehicle for ophthalmic use in the context of delivering proteins to the posterior segment of the eye. Previous studies of MOFs in ocular drug delivery have explored Fe-, Zr-, and Cu-based MOFs and were limited to release of a small molecule, brimonidine, finding that MOF particle characteristics make them suitable for ophthalmic drug delivery for the anterior segment [22–24]. However, drug delivery to the retina and the posterior segment of the eye is a challenge due to low efficacy and reduced bioavailability due the anatomical complexity of the tissue layers and the physiological fluid dynamics inside the posterior cavity as well as the ocular surface [3,4]. To address these challenges, lipid-based and polymeric nanoparticles have been used. These particles suffer from insufficient loading of proteins, inconsistent release kinetics, and reduced permeability through dense outer ocular tissue [25]. MOF-based delivery systems may improve these shortcomings. Recent studies have begun to explore ZIF-8 to treat ocular surface bacterial infections by loading and delivering antibiotics, either by instilling a singular suspension solution as an eye drop or incorporating ZIF-8 into contact lenses [26,27]. However, prior to the work reported herein, ZIF-8 has not been explored for topical ocular drug delivery to the retina.

Herein, we report on the use of ZIF-8 as a viable ocular drug delivery vehicle for proteins, like the anti-VEGF protein Ranibizumab ® used to treat wet AMD and DR. Compared to the MOFs previously studied, ZIF-8 can be fabricated in non-toxic solvents, provide a much larger surface area to encapsulate more cargo, and have better structural stability and biocompatibility when in contact with tissue. Here, we observed favorable release kinetics of a 42 kDa surrogate protein, ocular tissue compatibility, and ocular tissue permeability of both particle and surrogate protein cargo suggesting that ZIF-8 is well-positioned to deliver ocular therapeutics.

## Methods

### Synthesis of ZIF-8

The synthesis method is based off previously reported protocols that synthesized ZIF-8 in aqueous solutions at room temperature [28]. In brief, 1.16 g of zinc nitrate hexahydrate (Zn(NO_3_)_2_ ∙ 6H_2_O) (Sigma Alridch, St. Louis, MO, USA) was dissolved in 8 mL deionized water in a 15 mL tube. In a beaker, 22.70 g of 2-methylimidazole (Sigma Aldrich, St. Louis, MO, USA) was dissolved in 80 mL deionized water under stirring. The zinc nitrate solution was then added dropwise to the imidazole solution. The reaction mixture was left for 5 minutes under stirring at 600 rpm at room temperature and then collected by centrifugation and washed with deionized water 3 times. After washing, the material was freeze-dried for a minimum of 24 hours to ensure removal of excess water from pores at a temperature of ~ −57 °C and vacuum of ~30 mTorr (VirTis Benchtop Pro with Omnitronics^TM^ Freeze-Dryer, SP Scientific, Warminster, PA, USA). To encapsulate ovalbumin (ovaZIF-8) or fluoresceine (fluZIF-8), 1 g of ovalbumin (Research Products International, Mt. Prospect, IL, USA) or fluorescein sodium salt (Sigma Aldrich, St. Louis, MO, USA), respectively, was added to the imidazole solution prior to the addition of zinc nitrate.

### Material characterization

X-ray powder diffraction (PXRD) patterns of ZIF-8 and ovaZIF-8 were collected using a Bruker AXS D8 Discover powder diffractometer at 40 kV and a 40 mA for Cu-Kα (λ = 1.5406 Å) from 4 to 60° with a scan speed of 0.40 s/step and step size of 0.02°. Transmission electron microscopy (TEM) images were collected using a JEOL 1400-FLASH 120 kV TEM at the University of Pittsburgh Center for Biological Imaging. ZIF-8 and ovaZIF-8 were degassed and activated under vacuum for 12 hour at 105 °C and N_2_ isotherms were collected at 77 K on a Micromeritics 3flex gas adsorption analyzer. Infrared spectra were collected using a Nicolet iS50 FTIR Spectrometer with attenuated total reflectance sampling. Each sample was analyzed as a solid and scanned 64 times over a range of 1800−600 cm^-1^. The hydrodynamic diameter and zeta potential of the samples were determined using dynamic light scattering (DLS) on a Malvern Panalytical Zetasizer Nano ZS90. The measurements were carried out using a 1 mg/mL stock solution of ZIF-8 and ovaZIF-8 in ultrapure water at 25°C. For data processing, the refractive index of the particles was set to 1.4.

### Ovalbumin encapsulation and release studies

Ovalbumin quantification was carried out using a Pierce Micro Bicinchoninic Acid (BCA) Protein Assay kit (Thermo Fisher Scientific, Waltham, MA, USA). For loading assessments, aliquots of washing media were collected and analyzed. To calculate loading capacity, the total measured mass of protein was taken and divided by the total mass of particles.

For release tests, 10 mg of ovaZIF-8 (n = 3) and ZIF-8 (control, n = 1) were suspended in 500 µL of 1X phosphate buffer solution (PBS), pH 7.4 (Thermo Fisher Scientific, Waltham, MA USA). Samples were incubated on a rotator at 37°C. At predetermined points (8 hours on the first day, then daily for 7 days), samples were centrifuged at 6500 rpm for 10 minutes and the supernatant was collected. Fresh, pre-warmed PBS was added to the remaining sample, resuspended, and then returned to incubator to maintain sink-like conditions. After 7 days, samples were frozen until the end of the assessment. The blank-normalized amount of ovalbumin released was measured using a Pierce Micro BCA Protein Assay kit (Thermo Fisher Scientific, Waltham, MA USA).

### Ocular irritation studies

#### Hen’s Egg Test on Chorioallantoic Membrane (HET-CAM).

Fertilized single comb white leghorn hen eggs (n = 4) were obtained from Texas A&M University Poultry Science (College Station, TX, USA) and incubated for 9 days at 38 °C and 60% humidity in an automatic egg rotator with the pointy end facing down. On day 9, an egg candler was used to observe embryo formation in a dark room. Underdeveloped eggs were discarded. A 1 cm diameter circle was drawn on the outline of the air sac and a round grinding attachment on a rotary tool (Dremel, Walnut Ridge, AR, USA) was used to carefully cut and remove the eggshell to expose a thin protective membrane. Canned air was used to remove excess debris. The inner membrane was wetted with 500 µL of 0.9% NaCl solution and returned to the incubator for 30 minutes. The 0.9% NaCl solution was removed using a transfer pipette and curved forceps were used to carefully remove the inner membrane and expose the chorioallantois membrane (CAM). An image was taken to note any defects and characteristics of the CAM before application of samples. For assessment of ocular irritation, 0.9% NaCl and 0.1N NaOH were used as negative and positive controls. This procedure was repeated for powder samples of ZIF-8, ovaZIF-8, Zinc nitrate, and 2-methylimidazole. All powder samples were suspended in 0.9% NaCl at a concentration of 30 mg/mL. Controls of 300 µL and testing material were added to the CAM and pictures were taken at 30 seconds, 2 minutes, and 5 minutes. Images were masked and scoring was determined by the appearance of lysis, hemorrhaging, and/or coagulation. All materials were evaluated based on cumulative scoring.

#### Bovine Corneal Opacity and Permeability test (BCOP).

Freshly enucleated bovine eyes (n = 4 per group) were obtained overnight from Pel-Freez Biologicals (Rogers, AR, USA) and inspected for corneal damage. Bovine eyes with damage were discarded. Eyes were placed in small aluminum weigh dishes and incubated in a 37°C water bath for 10 minutes. A silicon O-ring was placed over the center of the cornea, and the corneal surface was wetted with 150 µL 0.9% NaCl solution. After 5 minutes, the solution was removed. The control and testing material were prepared and applied similarly as in the HET-CAM assessment. The materials were applied on the corneal surface for 30 seconds then rinsed off with 0.9% NaCl solution and incubated in a water bath for 10 minutes. Then, a fluorescein strip (Amcon, St. Louis, MO, USA) was applied to the corneal surface and illuminated with a blue cobalt light. Images were taken before and after, and changes in corneal opacity and fluorescein penetration were used to determine corneal irritation by the scoring guidelines outlined in Organization for Economic Cooperation and Development (OECD) Test No. 4327. Scores were determined via masked images taken during assessment and categorized based on cumulative scoring.

#### Ocular permeability.

Freshly enucleated porcine eyes were obtained the same day from a local abattoir (Thoma Meat Market, Saxonburg, PA, USA) with the optic nerve intact. Excess muscle and fat were removed with dissecting scissors. An incision was made at the corneal limbus to separate the anterior segment from the posterior segment of the eye. The full thickness cornea and vitreous body were discarded, leaving the retina-choroid-scleral complex.

Permeation experiments were conducted on Franz-type diffusion cells with a surface area of 0.64 cm^2^ (PermeGear Inc., Hellertown, PA, USA) using described procedures [29–31]. The dissected tissue (n = 3) was placed retina-side towards the receptor chamber and sealed well with the donor chamber to prevent sample leakage. The receptor chamber was filled with 5 mL of water and pre-heated to 37°C for 15 min using a water circulator. The receptor chamber was magnetically stirred to avoid any boundary layer effect. The donor chamber contained 500 µL of water with 10 mg fluZIF-8. Aliquots (300 µL) were collected from the receptor chamber immediately after filling the donor chamber, and then every hour for 5 hours. After each collection, the receptor chamber was refilled with an equal volume (300 µL) of fresh water. The amount of fluZIF-8 permeating through the sclera was determined by measuring the fluorescence in each aliquot and comparing it to an external calibration curve. Fluorescence was measured using a SpectraMax M4 microplate reader (Molecular Devices, Sunnyvale, Cal; ex = 486 nm, em = 519 nm).

At the end of the study, porcine eyes were washed once with water and then fixed in 4% paraformaldehyde (PFA) overnight at room temperature. The eyes were washed in 1X PBS to remove excess PFA, cut to only visualize the section that was exposed to the fluZIF-8 particles, then set in optimal cutting temperature (OCT) compound for cryo-sectioning. Tissue sections were cut at a thickness of 30 µm and imaged using a confocal microscope.

### Statistical analysis

HET-CAM and BCOP results were analyzed using a Kruskal-Wallis test and Dunn’s post-hoc analysis compared to 0.9% Saline. Ocular permeability (n = 3) was analyzed using the student’s t test and considered statistically significant when p-values were less than 0.05. Graphs were generated and statistical analyses were performed using GraphPad 10.

## Results

### Material characterization

#### Physical particle properties.

**Fig 1:**
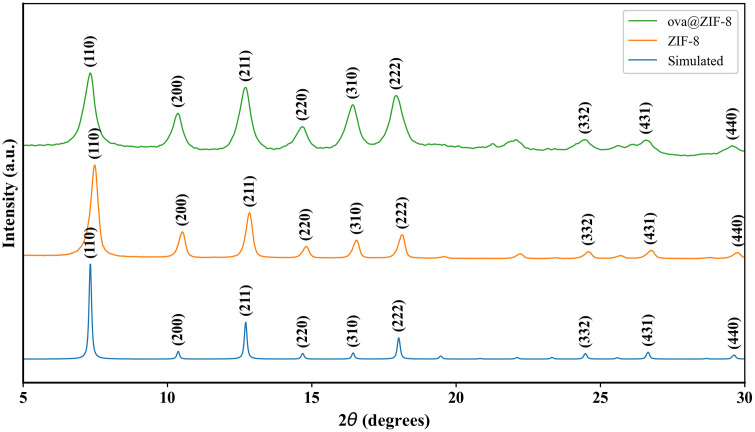
PXRD spectra of ZIF-8 and ovaZIF-8 compared to the spectra of simulated ZIF-8. Peaks observed at 2θ = 7°, 15°, and 30° are characteristic peaks of ZIF-8 and are conserved across the different samples.

**Fig 2:**
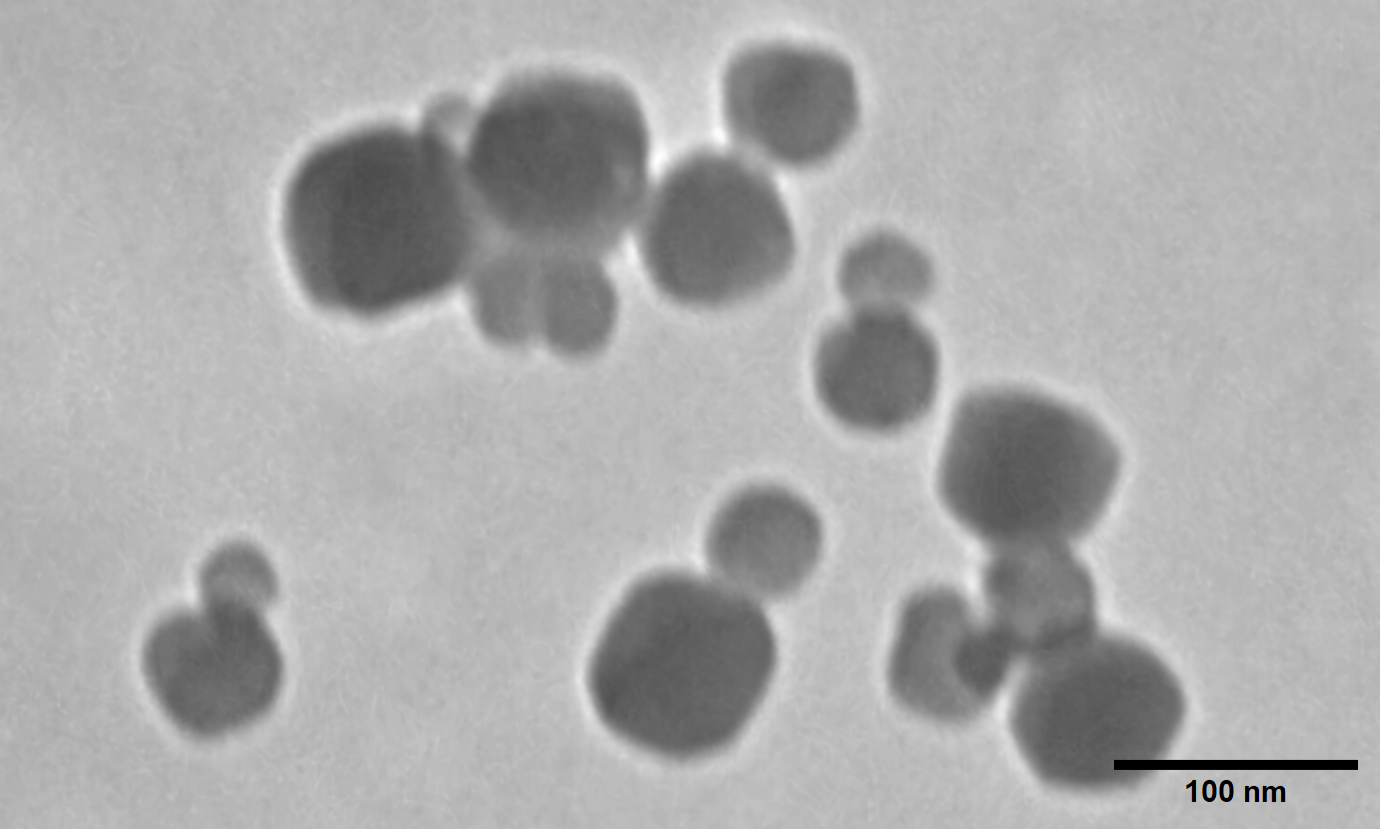
TEM image of ovaZIF-8. The particles exhibit dodecahedral morphology with diameters ranging from 50 to 100 nm. Scale bar = 100 nm.

PXRD was used to analyze the crystalline structure of ZIF-8. Crystallinity, phase purity, and unit size were determined from the spectra produced. As our studies used an aqueously synthesized ZIF-8 protocol, confirmation of typical structure was important. Diffraction peaks for ZIF-8 (CCDC #602542)appear at 2θ = 7.4°, 10.4°, 12.8°, 14.7°, 16.5°, 18.0°, 24.5°, 26.6°, and 29.7° which correspond to (110), (200), (211), (220), (310), (222), (332), (431), and (440) incident planes on the crystal surface [28]. The PXRD spectra of powder ZIF-8 and ovaZIF-8 are shown in Figure 1. The overlap of the spectral peaks not only confirms fabrication of structurally correct ZIF-8 but also phase purity of the material as a solid crystalline structure. No discernable differences are seen in the ovaZIF-8 spectra indicating that ovalbumin encapsulation did not affect the crystalline structure during fabrication. TEM further confirms the crystalline structure of ZIF-8 and ovaZIF-8. MOF sizes range between 50−100 nm and the characteristic dodecahedral morphology can be observed (Figure 2). DLS results showed a hydrodynamic diameter of 429 ± 107.5 nm and 525 ± 87.77 nm, zeta potentials of 17.1 ± 1.75 mV and −29.4 ± 0.74 mV, and polydispersity index (PDI) of 0.707 ± 0.130 and 0.475 ± 0.159 for ZIF-8 and ovaZIF-8, respectively.

#### FTIR Spectroscopy.

**Fig 3:**
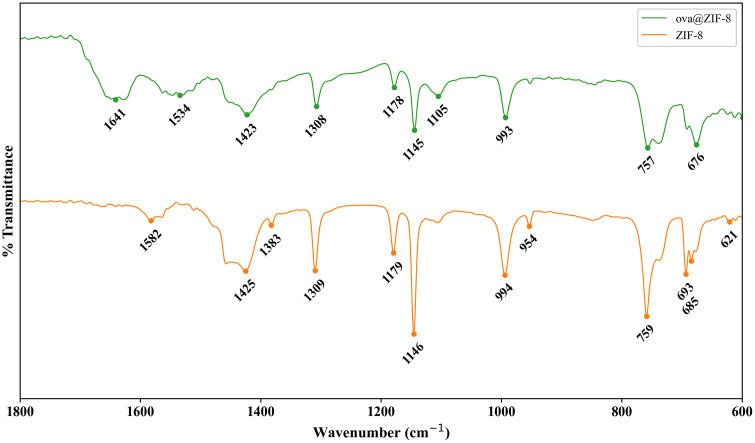
FTIR spectra of ZIF-8 and ovaZIF-8. Peaks observed at ~1300 cm^-1^, ~ 1145 cm^-1^, ~ 995 cm^-1^ correspond to correct ZIF-8 composition and high purity. Peak broadening at ~1750 cm^-1^ indicates successful encapsulation of ovalbumin.

We used FTIR spectroscopy to confirm the structure of ZIF-8 and evaluate the impact of ovalbumin loading [32]. We additionally evaluated the composition of our ZIF-8 compared to those synthesized via a non-aqueous method [17]. In its activated-dry powder form, vibrational spectral peaks of ZIF-8 appear at ~1600 cm^-1^ (C = N stretching), 1300−1500 cm^-1^ (imidazolate ring stretching), 1250−900 cm^-1^ (in-plane bending), and 800−600 cm^-1^ (out-of-plane bending) [33–35]. The FTIR spectra of powder ZIF-8 and ovaZIF-8 are shown in Figure 3. The corresponding spectra shows expected peaks and confirms the successful fabrication of ZIF-8. Peak broadening in the ovaZIF-8 spectra indicates successful ovalbumin encapsulation [28,33]. The consistent vibrational peaks before and after encapsulation indicate that the encapsulation of ovalbumin did not affect the overall composition of ZIF-8.

#### Gas adsorption/desorption.

**Fig 4:**
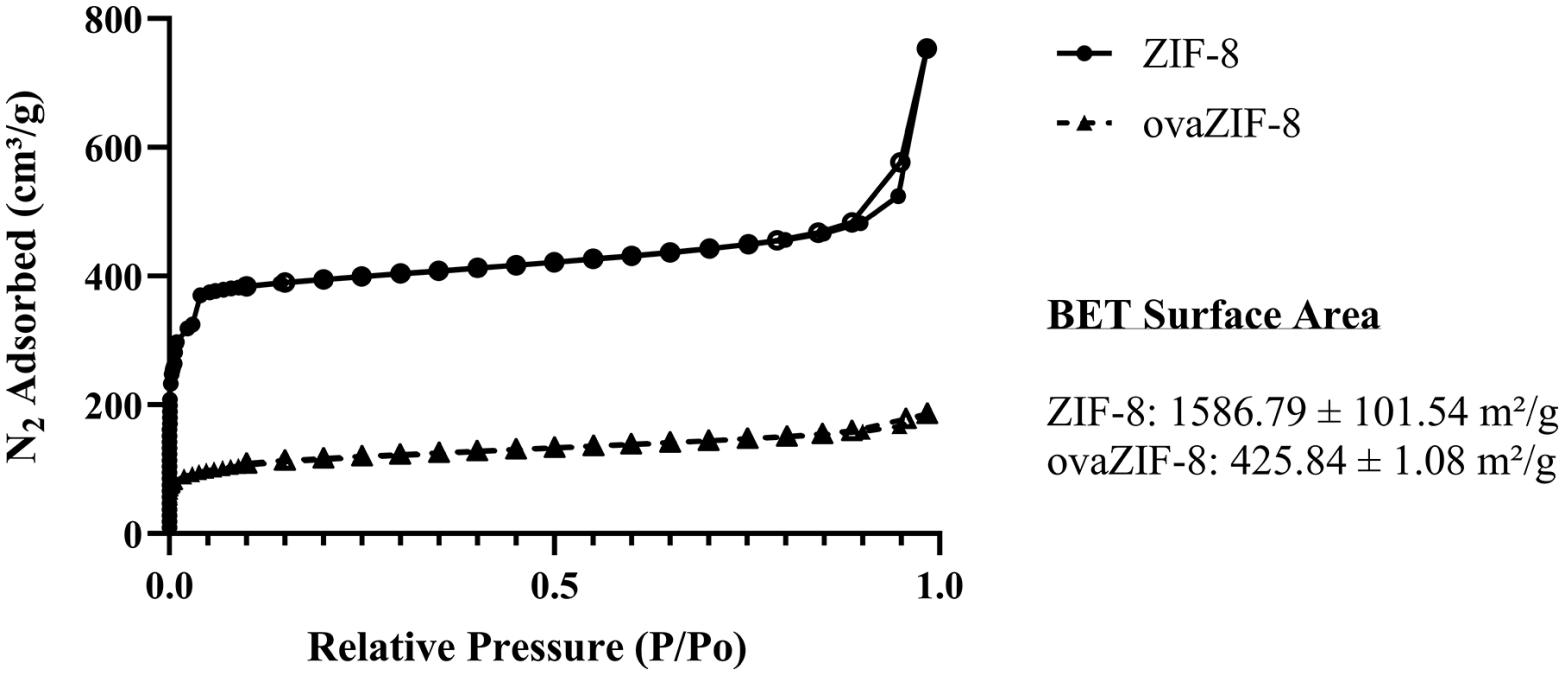
Nitrogen adsorption-desorption isotherms of ZIF-8 and ovaZIF-8. BET surface areas were 1586.79 m²/g for ZIF-8 and 425.84 m²/g for ovaZIF-8.

The nitrogen adsorption-desorption isotherms of ZIF-8 and ovaZIF-8 at 77 K are shown in Fig 4. The gas adsorption isotherms were used to further analyze the crystalline structure by measuring the internal porosity and surface area of the material. ZIF-8 typically demonstrates a type 1 isotherm, characterized by rapid absorption at low relative pressure and a horizontal plateau typical of microporous solids, with a second uptake indicating the existence of meso- and macro-porosity. Using the BET surface area equation [36], the surface area was determined to be 1586.79 m^2^/g and 425.84 m^2^/g for ZIF-8 and ovaZIF-8, respectively. This ~73% decrease in surface area indicates that ovalbumin has been encapsulated into the pores of the MOF, thus preventing N_2_ deposition. These results confirm the successful encapsulation of ovalbumin within the pores of ZIF-8.

#### Ovalbumin Encapsulation and Release Studies.

**Fig 5:**
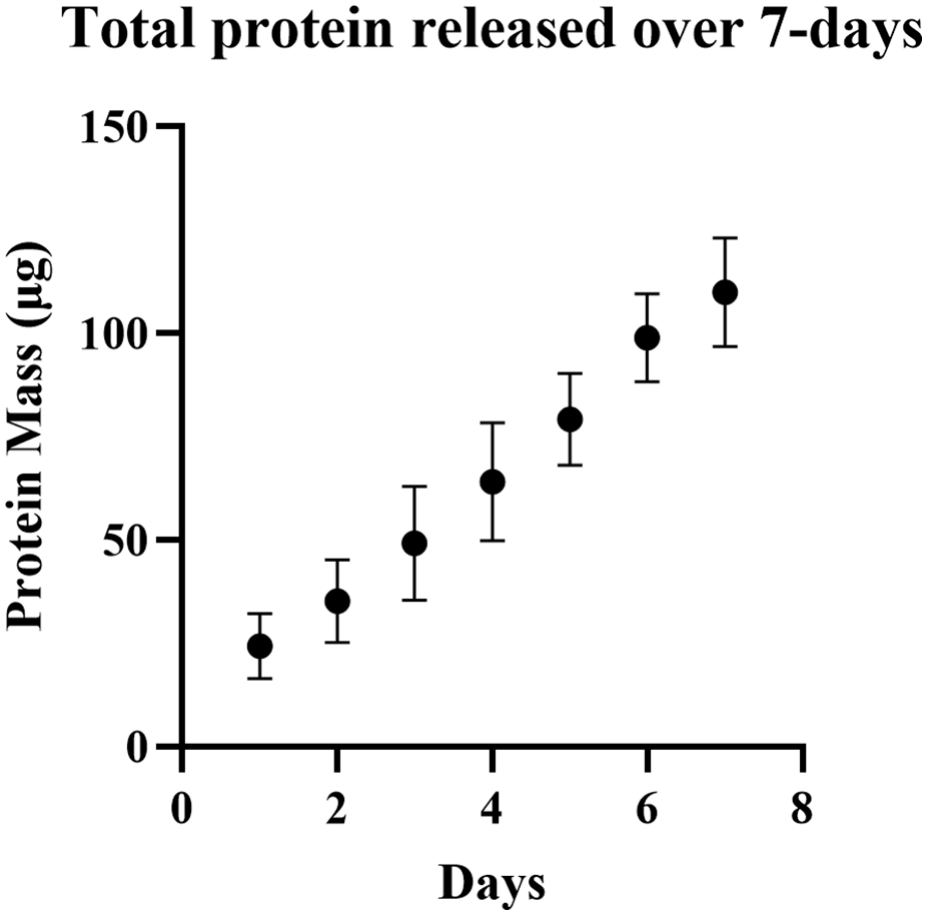
*In vitro* release profile of ovalbumin from ovaZIF-8 in 1X PBS, pH 7.4. Data represent mean ± standard error of the mean (n = 3).

For our release experiments, nanoparticles were submerged in 37°C 1X PBS, pH 7.4. Previous studies have shown that in these conditions ZIF-8 degrades more quickly at the nanoscale and forms zinc phosphate species, releasing encapsulated cargo [37]. To simulate the physiological environment of the ocular surface, we immersed 10 mg of ZIF-8 and ovaZIF-8 nanoparticles in 37°C 1X PBS, pH 7.4 and analyzed the *in vitro* release media over 7 days to determine the release kinetics and concentration accumulation. Over this period, we observed that our ovaZIF-8 demonstrated a controlled slow-release mechanism whereby approximately 200 µg of ovalbumin was cumulatively released over 7 days (Fig 5). The loading capacity for ovaZIF-8 was measured to be 0.4613 gram of ovalbumin per gram of particle (g/g). As a result, a 10 mg sample of ovaZIF-8 had 4.61 mg of ovalbumin encapsulated. While ZIF-8 particles have been used in other applications of drug delivery [22,38,39], to our knowledge this is the first application of a protein-loaded ZIF-8 for use in ocular drug delivery.

### Ocular irritation studies

#### HET-CAM & BCOP.

**Fig 6:**
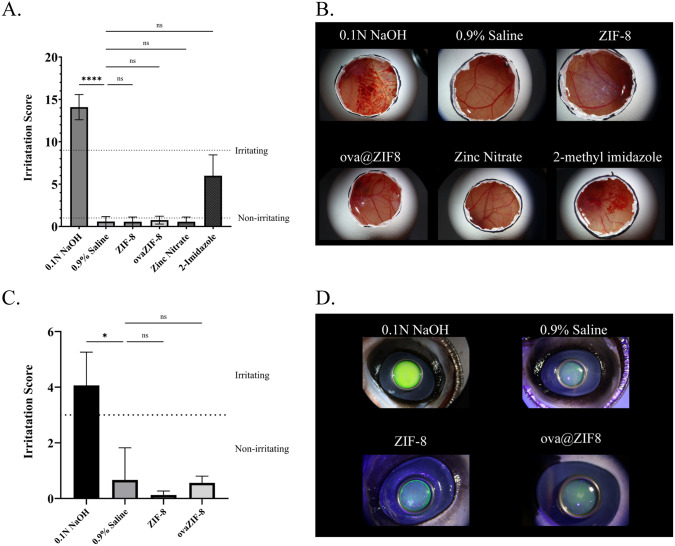
HET-CAM and BCOP irritation scores for ZIF-8, ovaZIF-8, fabrication components, and controls. **(A)** HET-CAM scores of ZIF-8 and ovaZIF-8, (B) representative HET-CAM images at 5 minutes after application of material. **(C)** BCOP scores of ZIF-8 and ovaZIF-8; (D) representative BCOP images at 2 minutes after application of material. Statistical analysis was performed using the Kruskal-Wallis test and Dunn’s post-hoc analysis compared to 0.9% Saline (p < 0.05 *, p < 0.001 ****).

To assess the irritancy of ZIF-8 and its components, we performed the HET-CAM and BCOP tests. The HET-CAM experiment was used to evaluate damage to microvasculature and mucous membranes of the ocular surface, while the BCOP experiment was used to assess damage to corneal tissue and function via a semi-quantitative scoring method based on observations over time concurrent with established tables and grouped into “non-irritating” and “severe irritating” categories [40,41]. For the HET-CAM assessment, aggregate scores demonstrated mild membrane irritation by ZIF-8 and ovaZIF-8 (Figs 6A and 6B). The BCOP assessment confirms HET-CAM observations with ZIF-8 and ovaZIF-8 showing no to slight damage to corneal tissue and function (Figs 6C and 6D).

#### Ex Vivo release and particle penetration.

**Fig 7:**
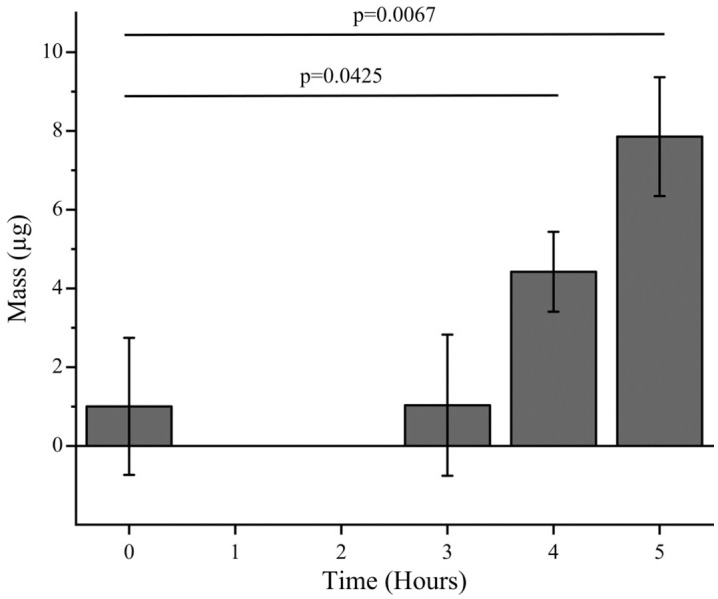
Porcine sclera permeability of fluorescein-loaded ZIF-8 (fluZIF-8) over 5 hours. Data are shown as mean ± standard deviation (n = 3).

**Fig 8:**
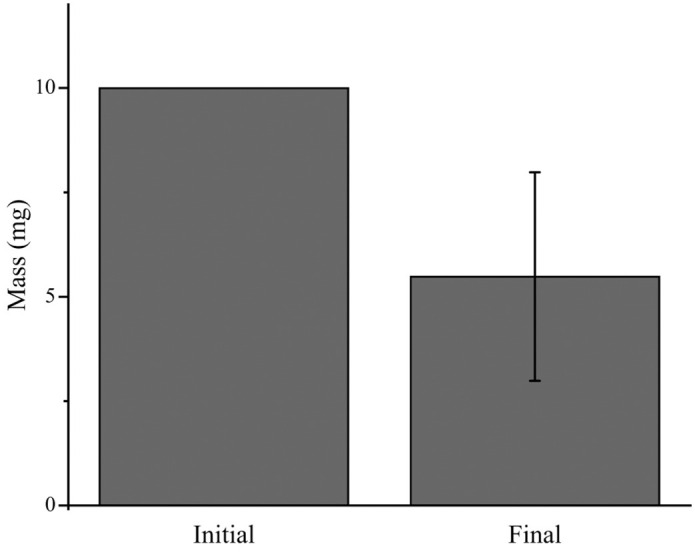
Remaining fluZIF-8 mass in the donor chamber over 5 hours after trans-scleral diffusion. Data are expressed as mean ± standard deviation (n = 3).

**Fig 9:**
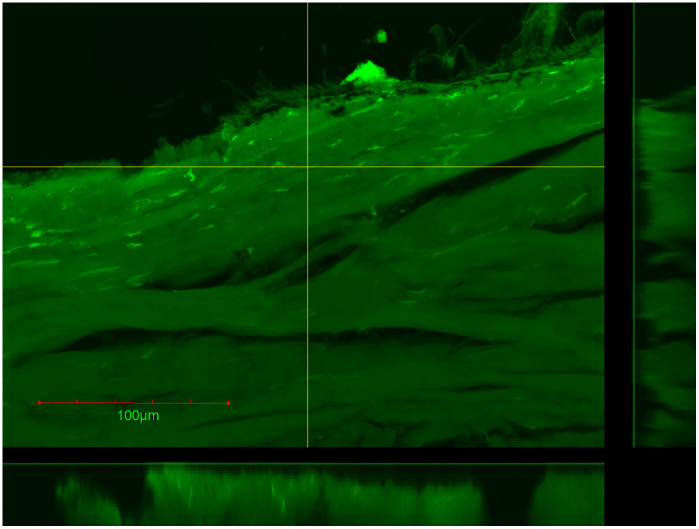
Confocal fluorescence imaging of sclera after 5-hour exposure of fluZIF-8. Punctate can be seen near the contact surface of suggesting uptake of fluZIF-8 into the collagen matrix. Scale bar = 100 µm.

For facile detection, ZIF-8 was loaded with fluorescein dye. Initially, 10 mg of fluorescein-loaded ZIF-8 (fluZIF-8) was suspended in 500 µL of water and deposited into the donor chamber of the Franz cell. Aliquots were collected from the receptor chamber every hour over a 5-hour period, and the amount of permeated ZIF-8 was determined by measuring the fluorescein fluorescence in each aliquot. While we observed a statistically significant increase in fluorescein fluorescence after 4 hours, the raw fluorescence values were small (~4 µg), suggesting that <1% of the deposited ZIF-8 had permeated through the tissue into the receptor chamber (Fig 7). In addition, after measuring the amount of fluorescence in the donor chamber, we calculated a 50% decrease in fluZIF-8 mass (Fig 8) which likely suggests that ZIF-8 can enter the sclera and transport cargo through this pathway. Fig 9 shows particle punctate near the contact surface between the fluZIF-8 particles and the sclera suggesting uptake into the collagen matrix.

## Discussion

In this study, we explored the use of ZIF-8 as a carrier for ovalbumin, a surrogate for the anti-VEGF fragment antibody Ranibizumab®, in ocular drug delivery. Previous ocular drug delivery studies using MOFs have focused on the delivery of brimonidine, a small molecule used to treat glaucoma. Those studies found that MOFs have loading capacities above 50 wt% and can effectively deliver brimonidine to the anterior segment of the eye with controlled release kinetics over days [22,23]. However, topical drug delivery to the retina and the posterior segment of the eye is still a challenge resulting in low efficacy and reduced bioavailability [3,4]. Herein, we discuss the results of our exploratory study on the use of ZIF-8 as an ocular drug delivery vehicle to the retina and posterior segment. Specifically, we examined the physical and chemical properties of ZIF-8 encapsulated with ovalbumin protein (42 kDa). We additionally examined the interactions of ZIF-8 and its components on the ocular surface, and the ability of ZIF-8 to deliver ovalbumin to the posterior segment of the eye.

ZIF-8 are a zeolitic imidazolate framework composed of Zn^2+^ metallic nodes linked together by 2-methylimidazole to form a nano-sized, 3-D crystalline structure with a large surface area and heterogenous porosity [28]. ZIF-8 shows moderate stability in water at neutral pH [42,43]. In our results, we observed consistent composition of ZIF-8 and ovaZIF-8 by PXRD and FTIR. Compared to the simulated ZIF-8 PXRD spectral peaks from the Cambridge Crystallographic Data Center (https://www.ccdc.cam.ac.uk/structures/), we observed some peak broadening in our measurements (Fig 1). We attribute this broadening to the impact of encapsulation with ovalbumin. The most intense peak at θ = ~7.4° (110) is a hallmark of ZIF-8 crystallography corresponding to interplanar spacing due to the large cages and pore channels in the structure [44]. The retention of this peak in our ovaZIF-8 spectrum, in addition to the lack of crystallographic changes, confirms that our ZIF-8 retains its crystal structure even after loading with ovalbumin. Similarly, our FTIR spectra (Fig 2) showed the characteristic imidazolate ring stretching and bending observed between 1500−600 cm^-1^ before and after loading with ovalbumin. Peaks at ~1650 cm^-1^ and ~1500 cm^-1^ in our ovaZIF-8 samples are attributed to the ovalbumin protein, indicating successful encapsulation. The successful encapsulation of ovalbumin in ZIF-8 was further confirmed by the adsorption isotherms under nitrogen, where a 73% reduction in BET surface area was observed (Fig 3), in addition to a shift in particle size and zeta potential.

We additionally characterized particle size of our ZIF-8 using DLS and TEM. Our results demonstrate a mismatch between TEM and DLS measurements, with DLS showing a roughly 300% increase in particle diameter of ZIF-8 and ovaZIF-8 than that observed by TEM. This is expected [45], since unlike TEM, DLS measures the hydrodynamic radius of a particle, and thus includes a shell of water that increases the apparent diameter, explained by the stability of ZIF-8 in water [28,42]. Furthermore, the high polydispersity indicates potential particle aggregation in suspension. Because ZIF-8 degradation and cargo release are dependent on surface area, particle aggregation may alter release kinetics and may influence size-dependent tissue permeability [37,46,47].

ZIF-8 release is typically considered stimuli-responsive, and its stability depends on the chemical composition of the environment. While ZIF-8 has been shown to be moderate stable in water at ambient temperature [42], it degrades in anionic and acid solutions [37,48,49]. In our studies, we used 1X PBS (pH 7.4) to mimic the tear film environment. Human tear film is composed of a complex mixture of proteins, lipids, and ions in a water-based, neutral pH environment that keeps the anterior segment dry and clean [50–52]. Artificial tears are like PBS but have a higher phosphate concentration. With this in mind, we simulated the release kinetics of ZIF-8 in 1X PBS (pH 7.4) at 37 °C and observed a linear release profile over a 7-day period with approximately 200 µg of ovalbumin cumulatively released out of a starting amount of 4.61 mg of encapsulated ovalbumin. This corresponds to approximately 4% of encapsulated ovalbumin being released (Fig 5), while another 4% was released in the following 60 days. We hypothesize this is a result of the degradation byproducts reforming into a zinc-phosphate particle via electrostatic interactions that re-encapsulate a fraction of the ovalbumin that was released, as observed in a previous study by Velasquez-Hernandez et. al [37]. Previous encapsulation of proteins in ZIF-8 have shown lower encapsulation (~0.150 g/g) and quick release in PBS pH 7.4 of over 72% in only 24 hours [48]. In the context of therapeutic potential, the limited fractional release of ovalbumin may be clinically relevant. For AMD and DR, a typical intravitreal injection of ranibizumab is approximately 300−500 µg administered monthly. We demonstrate the cumulative release of ~200 µg of ovalbumin over 7 days is within the same order of magnitude as clinically used doses, and we observe much of the remaining ovalbumin is encapsulated within the particle suggesting a sustained-release depot rather than rapid burst release typically associated with polymeric formulations [53,54]. This supports the potential relevance of ZIF-8 physiochemical properties for maintaining therapeutic levels and mitigating risks of bolus dosing.

Previous studies on ZIF-8 biocompatibility have shown low risk in intranasal delivery [55], cancer therapies [56,57], and tissue regeneration [58,59]. To support literature claims of ZIF-8 biocompatibility, the byproducts of the degradation of ZIF-8 were analyzed to ensure tolerance with the ocular surface and tissue. Irritation to the ocular surface presents risks of infiltration and inflammation of the inner compartments and damage to retinal tissue [60]. As seen in Fig 6, ZIF-8 and ovaZIF-8 did not irritate the ocular surface. Zinc is an abundant ion in the human body and plays key roles as an antioxidant in treating age-related conditions and helping maintain ocular surface stability [61,62]. However, 2-methylimidazole did show moderate irritation (Fig 6A). This could be a concern if 2-methylimidazole is presented as a degradation byproduct and will be explored in future in vivo assessments. Previous studies on MOF use in ocular drug delivery have solely used cellular viability studies with corneal epithelial cells [22,23]. However, these studies do not demonstrate the larger effects of material-host interaction that is necessary for translational studies.

Porcine sclera is commonly used in trans-scleral permeability experiments due to its structural and functional similarities to the human sclera. Although porcine sclera is twice as thick as the human sclera, the histology and collagen structure are comparable [63]. For ZIF-8 to be effective as a delivery vehicle for the treatment of retinal disease, it must be able to permeate through the sclera and accumulate in choroidal or retinal tissue. In previous trans-scleral permeability studies, liposomes and polystyrene nanoparticles of similar or smaller size have shown to pass through the scleral mainly due to their size, composition, and surface charges [64,65], but calculated permeability amounts show around 1% of the applied amount is found in the tissue [46]. We aimed to explore two questions with this experiment: does ZIF-8 permeate through the ocular tissue and are loaded delivery agents released into the sclera. Previous studies have shown that ZIF-8 can penetrate through the corneal epithelium and skin, both of which are much denser and more impermeable than the sclera [66]. Additionally, ZIF-8 can be taken up intracellularly to bypass limiting membranes or barriers [27].

We evaluated the permeation and delivery of loaded agents into ex vivo porcine eyes using ZIF-8 loaded with fluorescein salt. We observed some permeation of fluZIF-8, indicating our ZIF-8 can pass through the scleral tissue. However, the permeation we observed was smaller than what we expected based on the reported permeability of ZIF-8. We hypothesize that this lower permeability is likely due to the thickness of the porcine ocular tissue and particle aggregation in the bulk solution. The smaller permeability is not necessarily detrimental to translating into in vivo studies since we are interested in delivering therapeutics to the sclera, and thus the loaded ZIF-8 do not have to pass through all layers of the ocular tissue. To evaluate how much fluZIF-8 reached the sclera, at the conclusion of the experiment, we measured the amount of fluZIF-8 that remained in the donor chamber and thus did not penetrate any tissue. We observed that only ~50% of the deposited fluZIF-8 remained in the donor chamber, indicating that up to 50% of the fluZIF-8 could have penetrated the tissue. To evaluate release into the sclera, we took histological images of the scleral tissue at the conclusion of the experiment. While we observed some evidence of fluorescence in the sclera, it was difficult to distinguish fluZIF-8 from the autofluorescence of the ocular tissue, and thus further experimentation is needed to fully understand where loaded agents are released in the eye. Future experiments will further confirm ex vivo release to the sclera utilizing thinner tissue that is more comparable to the human sclera, as well as in vivo assessments to investigate ZIF-8 particle diffusion behaviors and cargo disposition. We will additionally utilize a hydrophobic internal probe with a fluorescently tagged imidazole linker to visualize colocalization of cargo and particle in posterior segment ocular tissue.

## Conclusion

This study demonstrates the viability of ZIF-8 as a novel platform for topical ocular drug delivery. By leveraging their high loading capacity, biocompatibility, and pH-responsive release kinetics, ZIF-8 offers significant advantages over conventional delivery methods such as lipid-based or polymeric nanoparticles. Characterization confirmed the structural integrity and encapsulation efficiency of ZIF-8 for model drugs such as ovalbumin and fluorescein. Sustained ovalbumin release over seven days suggest the potential of ZIF-8 to carry large quantity of cargos and demonstrate cumulative amounts similar to clinically used doses.

Ex vivo permeability studies suggest that fluorescein-loaded ZIF-8 can penetrate the scleral barrier, suggesting their ability to deliver targeted therapeutics to posterior ocular tissues. Moreover, ocular irritation assessments demonstrated that ZIF-8 is non-toxic and well-tolerated, further supporting its suitability for ophthalmic use. Future research will focus on optimizing ZIF-8 formulations for in vivo studies to evaluate long-term efficacy and safety. This work lays a critical foundation for advancing MOF-based drug delivery systems in treating ocular diseases.

## Supporting information

S1 Fig TEM images at different magnifications. (DOCX)

S2 Fig DLS plots of ZIF-8 and ovaZIF-8. (DOCX)

S3 Fig Calibration curve of permeability by fluorescence. (DOCX)
