# Supplementary material for: Zeolitic imidazolate frameworks (ZIF-8) as a carrier in a topical ocular delivery system for the treatment of ocular diseases
Source: PLoS One. 2026 Apr 21;21(4):e0346473. doi: 10.1371/journal.pone.0346473 (PMC13098936; doi:10.1371/journal.pone.0346473)
Supplement: S1 Fig — (DOCX) [file pone.0346473.s001.docx]

**Figure S1.1 Figure S1.2**


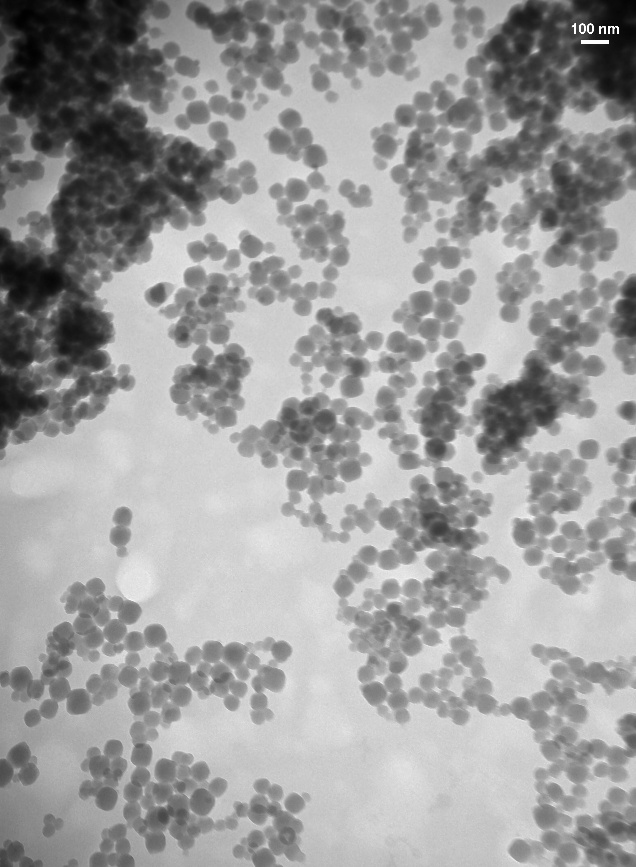

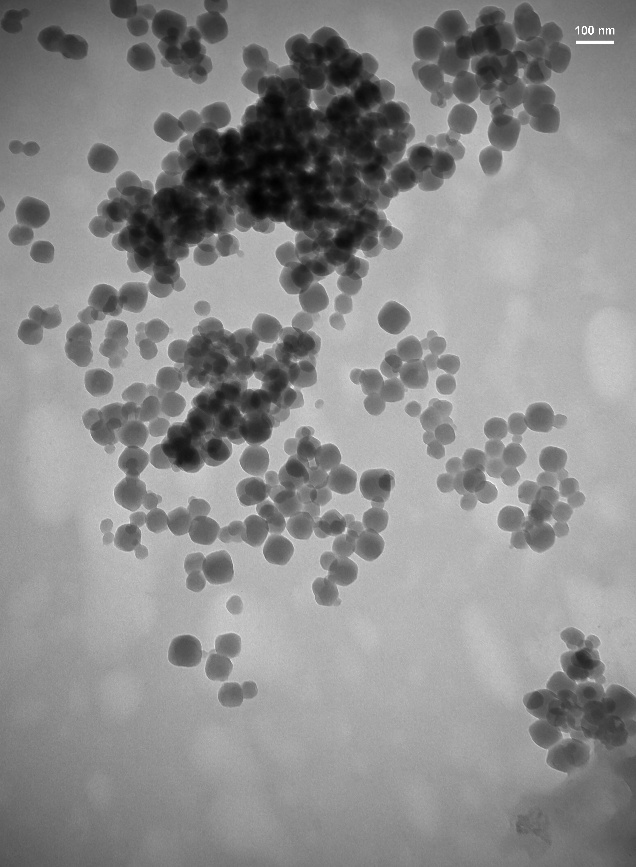


Figure S1.1 and Figure S1.2 demonstrate the size and morphology of ZIF-8 nanoparticles at 100 nm and 500 nm, respectively.


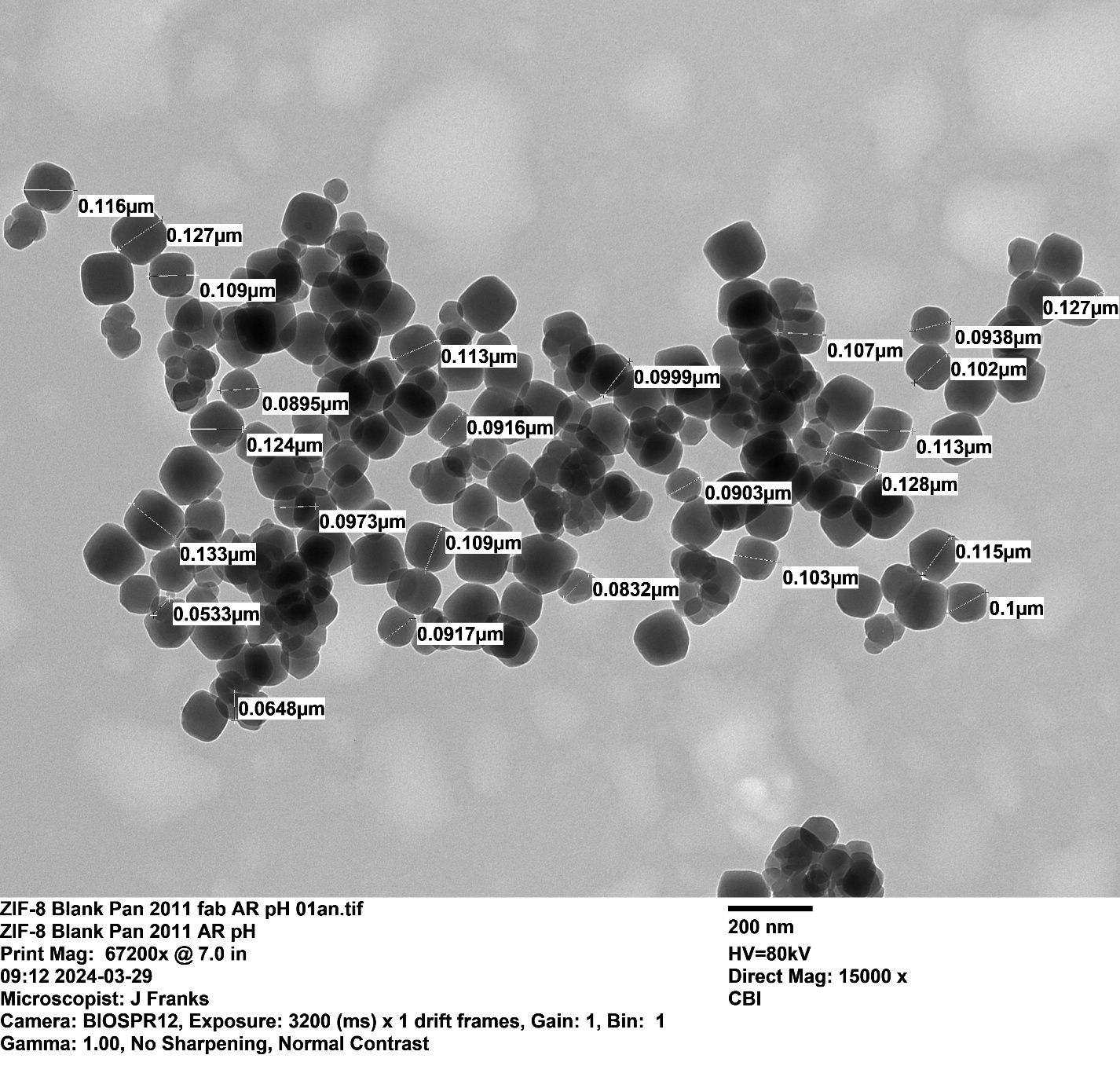


**Figure S1.3.** Annotated TEM image of ZIF-8 nanoparticles for size distribution measurements.


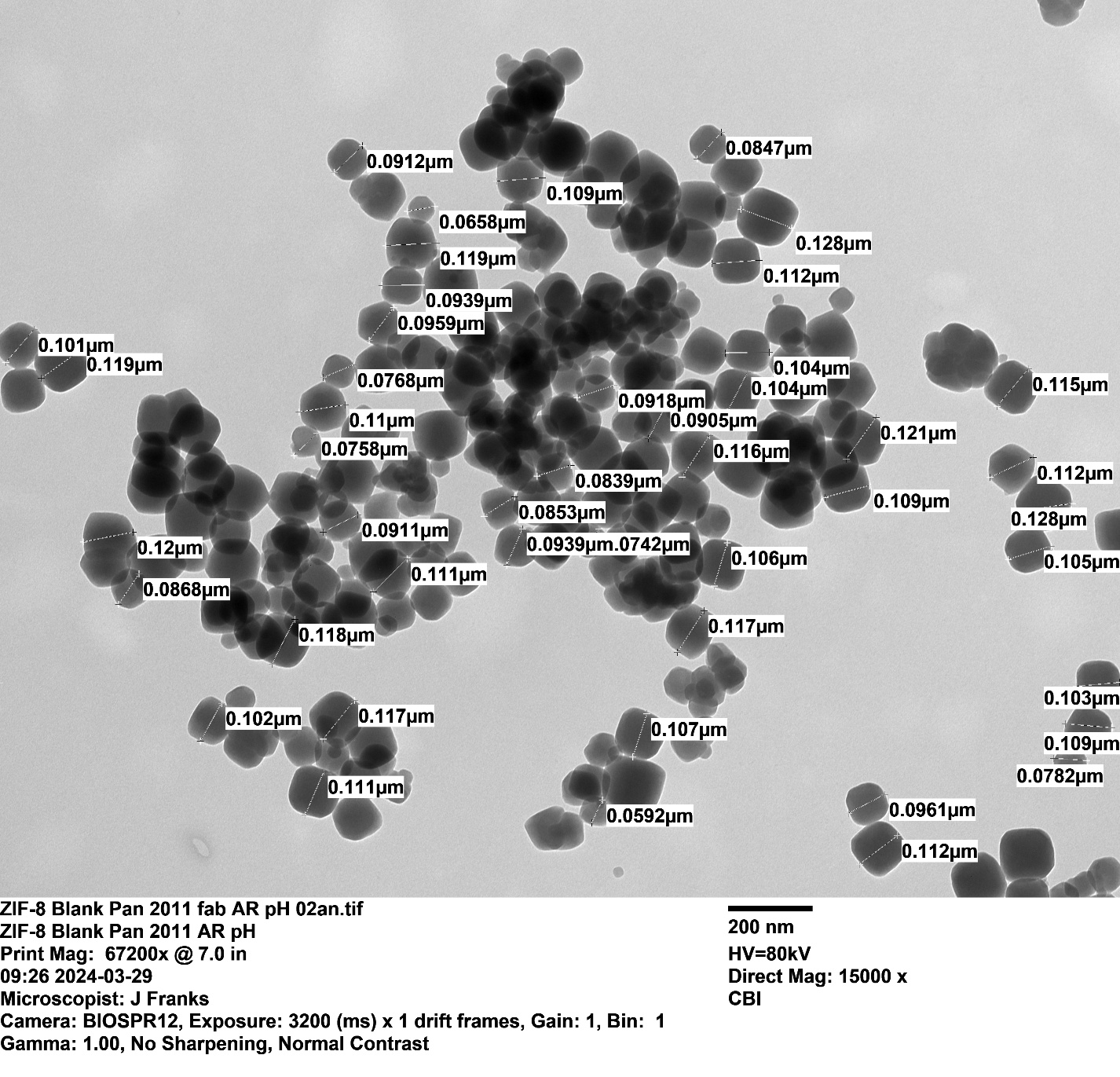


**Figure S1.4.** Annotated TEM image of ZIF-8 nanoparticles for size distribution measurements.


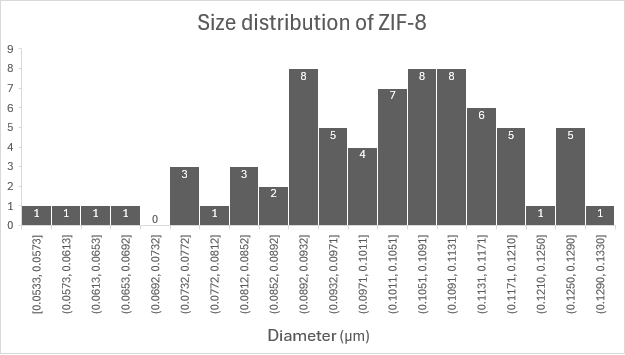


**Figure S1.5.** Histogram of size distributions from TEM images.
