# Supplementary material for: Zeolitic imidazolate frameworks (ZIF-8) as a carrier in a topical ocular delivery system for the treatment of ocular diseases
Source: PLoS One. 2026 Apr 21;21(4):e0346473. doi: 10.1371/journal.pone.0346473 (PMC13098936; doi:10.1371/journal.pone.0346473)
Supplement: S2 Fig — (DOCX) [file pone.0346473.s002.docx]

Ovalbumin ZIF-8 (ova@ZIF8) #1

**PARTICLE SIZE**

MEAN: 525, SD: 87.77, MAX: 623.5

X Intensity Record 3: 1 Record 4: 2 Record 5: 3

0.400 0.000 0.000 0.000

0.463 0.000 0.000 0.000

0.536 0.000 0.000 0.000

0.621 0.000 0.000 0.000

0.719 0.000 0.000 0.000

0.833 0.000 0.000 0.000

0.965 0.000 0.000 0.000

1.117 0.000 0.000 0.000

1.294 0.000 0.000 0.000

1.499 0.000 0.000 0.000

1.736 0.000 0.000 0.000

2.010 0.000 0.000 0.000

2.328 0.000 0.000 0.000

2.696 0.000 0.000 0.000

3.122 0.000 0.000 0.000

3.615 0.000 0.000 0.000

4.187 0.000 0.000 0.000

4.849 0.000 0.000 0.000

5.615 0.000 0.000 0.000

6.503 0.000 0.000 0.000

7.531 0.000 0.000 0.000

8.721 0.000 0.000 0.000

10.100 0.000 0.000 0.000

11.696 0.000 0.000 0.000

13.545 0.000 0.000 0.000

15.686 0.000 0.000 0.000

18.166 0.000 0.000 0.000

21.037 0.000 0.000 0.000

24.363 0.000 0.000 0.000

28.214 0.000 0.000 0.000

32.674 0.000 0.000 0.000

37.840 0.000 0.000 0.000

43.821 0.000 0.000 0.000

50.748 0.000 0.000 0.000

58.771 0.000 0.000 0.000

68.061 0.000 0.000 0.000

78.820 0.000 0.000 0.000

91.280 0.000 0.000 0.000

105.709 0.000 0.000 0.628

122.420 0.000 0.000 1.503

141.772 0.000 0.000 2.166

164.183 0.000 2.265 2.492

190.137 0.000 5.765 2.677

220.194 0.027 8.530 3.064

255.002 8.551 9.579 3.932

295.312 21.217 9.066 5.361

341.995 28.961 7.755 7.190

396.058 25.768 6.452 9.068

458.666 13.576 5.652 10.559

531.172 1.900 5.448 11.271

615.139 0.000 5.615 10.970

712.379 0.000 5.787 9.648

824.992 0.000 5.630 7.534

955.406 0.000 4.971 5.053

1106.435 0.000 3.849 2.715

1281.340 0.000 2.489 0.986

1483.893 0.000 1.220 0.119

1718.466 0.000 0.346 0.000

1990.119 0.000 0.000 0.000

2304.716 0.000 0.000 0.000

2669.043 0.000 0.000 0.000

3090.964 0.000 0.000 0.000

3579.581 0.000 0.372 0.000

4145.438 0.000 1.433 0.242

4800.746 0.000 3.043 0.930

5559.644 0.000 4.735 1.892

6438.508 0.000 0.000 0.000

7456.302 0.000 0.000 0.000

8634.988 0.000 0.000 0.000

10000.000 0.000 0.000 0.000

Ovalbumin ZIF-8 (ova@ZIF8) #2

**ZETA POTENTIAL**

MEAN: -29.4 mV; SD: 0.737, MAX: -28.8

ZIF-8 (blank) #1

**PARTICLE SIZE**

MEAN: 429.5, SD:107.5, MAX: 527.6

X Intensity Record 10: ZIF1-blank 1 Record 11: ZIF1-blank 2 Record 12: ZIF1-blank 3

0.400 0.000 0.000 0.000

0.463 0.000 0.000 0.000

0.536 0.000 0.000 0.000

0.621 0.000 0.000 0.000

0.719 0.000 0.000 0.000

0.833 0.000 0.000 0.000

0.965 0.000 0.000 0.000

1.117 0.000 0.000 0.000

1.294 0.000 0.000 0.000

1.499 0.000 0.000 0.000

1.736 0.000 0.000 0.000

2.010 0.000 0.000 0.000

2.328 0.000 0.000 0.000

2.696 0.000 0.000 0.000

3.122 0.000 0.000 0.000

3.615 0.000 0.000 0.000

4.187 0.000 0.000 0.000

4.849 0.000 0.000 0.000

5.615 0.000 0.000 0.000

6.503 0.000 0.000 0.000

7.531 0.000 0.000 0.000

8.721 0.000 0.000 0.000

10.100 0.000 0.000 0.000

11.696 0.000 0.000 0.000

13.545 0.000 0.000 0.000

15.686 0.000 0.000 0.000

18.166 0.000 0.000 0.000

21.037 0.000 0.000 0.000

24.363 0.000 0.000 0.000

28.214 0.000 0.000 0.000

32.674 0.000 0.000 0.000

37.840 0.000 0.000 0.000

43.821 0.000 0.000 0.000

50.748 0.000 0.000 0.000

58.771 0.000 0.000 0.000

68.061 0.000 0.000 0.000

78.820 0.000 0.000 0.000

91.280 0.000 0.000 0.000

105.709 0.000 0.000 0.670

122.420 0.000 2.634 4.913

141.772 35.539 19.994 11.365

164.183 48.836 34.446 16.971

190.137 15.625 30.553 19.160

220.194 0.000 12.373 17.105

255.002 0.000 0.000 11.925

295.312 0.000 0.000 5.978

341.995 0.000 0.000 1.634

396.058 0.000 0.000 0.000

458.666 0.000 0.000 0.000

531.172 0.000 0.000 0.146

615.139 0.000 0.000 1.178

712.379 0.000 0.000 2.470

824.992 0.000 0.000 3.039

955.406 0.000 0.000 2.411

1106.435 0.000 0.000 1.034

1281.340 0.000 0.000 0.000

1483.893 0.000 0.000 0.000

1718.466 0.000 0.000 0.000

1990.119 0.000 0.000 0.000

2304.716 0.000 0.000 0.000

2669.043 0.000 0.000 0.000

3090.964 0.000 0.000 0.000

3579.581 0.000 0.000 0.000

4145.438 0.000 0.000 0.000

4800.746 0.000 0.000 0.000

5559.644 0.000 0.000 0.000

6438.508 0.000 0.000 0.000

7456.302 0.000 0.000 0.000

8634.988 0.000 0.000 0.000

10000.000 0.000 0.000 0.000

ZIF-8 (blank) #2

**ZETA POTENTIAL**

MEAN: 17.1, SD:1.75, MAX: 18.6
