# Supplementary material for: Zeolitic imidazolate frameworks (ZIF-8) as a carrier in a topical ocular delivery system for the treatment of ocular diseases
Source: PLoS One. 2026 Apr 21;21(4):e0346473. doi: 10.1371/journal.pone.0346473 (PMC13098936; doi:10.1371/journal.pone.0346473)
Supplement: S3 Fig — (DOCX) [file pone.0346473.s003.docx]

**Figure S3.** Calibration curve of permeability by fluorescence.
